# Elemental imaging by Laser-Induced Breakdown Spectroscopy to evaluate selenium enrichment effects in edible mushrooms

**DOI:** 10.1038/s41598-019-47338-7

**Published:** 2019-07-25

**Authors:** Aline Pereira de Oliveira, Flávio de Oliveira Leme, Cassiana Seimi Nomura, Juliana Naozuka

**Affiliations:** 1 Universidade Federal de São Paulo, Departamento de Química, Diadema, 09972-270 Brazil; 2 Universidade Federal do ABC, Centro de Ciências Naturais e Humanas, Santo André, 09210-580 Brazil; 3 Universidade de São Paulo, Departamento de Química Fundamental, São Paulo, 05513-970 Brazil

**Keywords:** Imaging studies, Bioanalytical chemistry

## Abstract

Mushrooms are bioaccumulating organisms commonly used in selenium (Se) enrichment studies. However, the addition of Se in the culture medium may alter the distribution of other essential elements in the mushroom fruiting body. To evaluate the effects of the Se enrichment, Ca, Mg, and K distributions in pink oyster (*Pleurotus djamor*) and K and Mg distributions in white oyster (*Pleurotus ostreatus*) mushrooms were mapped by laser-induced breakdown spectroscopy (LIBS), which can be used at room temperature and requires minimal or no sample preparation. It was verified that Se enrichment favoured the accumulation of Ca in the lower part of the pink oyster mushroom and prevented the transport of this element to the edges and tops. The Se enrichment also altered the distribution of K and Mg, decreasing the numerical correlation between the K and Mg distributions (R² = 0.5871). In the white oyster mushroom, however, despite the changes in the morphological characteristics of the fruiting bodies after enrichment, there were generally nonsignificant differences in the K and Mg distributions between the control and the Se-enriched mushrooms.

## Introduction

The ideal food should contain all the macro and micronutrients required by humans. However, certain nutrients, primarily microelements, are not always available in foods, and are therefore of major importance for introduction into the diet through food re-education, drug supplementation, or the fortification/enrichment of foods. Food enrichment is the recommended intervention to address micronutrient deficiency, mainly in countries with high nutritional deficiencies. The addition of the appropriate compounds in industrial or processed foods has been used to reduce micronutrient deficiencies. Dairy products and cereals are commonly fortified, allowing the addition of micronutrients to children’s diets. Cultivated foods, such as fruits, vegetables, nuts, mushrooms, cereals, beans, and rice, grown in soils or media previously treated with the micronutrients of interest, is a promising alternative to fortification and an ally of the agricultural economy, culminating in the production of superfoods and functional foods^1^.

With these considerations in mind, the production of enriched edible mushrooms is particularly noteworthy in the world market. The consumption of edible mushrooms, primarily those of the genus *Pleurotus*, shows rapid expansion owing to its refined taste, high nutritional value, and medicinal properties^1^. These mushrooms are good sources of proteins, carbohydrates, vitamins, calcium, and iron. In addition, they are low in cost and have important medicinal effects, such as antitumorigenic properties, modulation of cholesterol levels, prevention of platelet aggregation in the arteries to avoid cardiovascular diseases, combating of the hepatitis C virus, and antioxidant and antibacterial properties^1^. Furthermore, the mushrooms are able to bioaccumulate essential elements in their fruiting bodies because they absorb nutrients from the substrate used as a growing medium, which is composed of an organic material such as sugarcane bagasse^2,3^. As a result of their high bioaccumulating potential and the scarcity of good sources of selenium for human health, mushrooms have been enriched with this essential element during growth stages that precede the formation of the fruiting bodies^2,4–7^.

Food enrichment allows for functional food production with high economic value. It favours the prevention or recovery of nutritional deficiencies, and therefore is the recommended intervention for localities where a high prevalence of nutritional deficiencies is found. The strategies commonly used to produce Se-enriched mushrooms is the addition of sodium selenite to the organic substrate used in cultivation^2,8,9^.

It is necessary, however, to evaluate whether the enriched food has the same morphological and chemical characteristics as the food grown under normal conditions. The enrichment may change the chemical composition and distribution of essential elements in the mushrooms^4,10^. For this reason, an evaluation of the distribution of essential elements is imperative to valorize the enriched foods, with the goal of describing and understanding the biological functions of the essential elements in mushrooms, as well as synergistic or antagonistic relationships between different elements in biochemical processes caused by enrichment. In order to obtain information about the absorption, translocation, and storage of essential elements in these hyperaccumulating organisms, analytical methods that allow to determine the total concentration of essential elements can be applied, however elemental mapping or imaging procedures could also be used to the same purpose, since that it is feasible, simply and rapid^11,12^.

Laser-induced breakdown spectroscopy (LIBS) is an emerging technique for elemental determination and mapping using spectra, analytical signals, or elemental concentrations^12–14^. The elemental mapping by LIBS presents several advantages, such as simplicity, ease of operation at room temperature and pressure, and minimal or no sample preparation^11,15^. These features make the LIBS-based images a promising and fast method for investigations of elemental distribution and mapping in different samples^12,16–20^.

Considering the importance and effects of the enrichment procedures of these hyperaccumulating organisms (mushrooms), the use of LIBS imaging is a valuable method to study the alteration to essential elements, such as calcium (Ca), magnesium (Mg) and potassium (K) that may occur during Se enrichment in pink (*Pleurotus djamor*) and white (*Pleurotus ostreatus*) oyster mushrooms.

## Results and Discussion

### Evaluation of the Se enrichment

The Ca, Mg, K, and Se amounts were determined by ICP OES after acid digestion of the two mushroom species and groups (control and enriched).

The LOD and LOQ were obtained in µg/g values, considering a sample mass of 30 mg and a final volume of 10 mL for the digested solutions. The LODs and LOQs ranged from 0.6 (Se) to 12.9 (K) µg/g and 5.8 (Se) to 128.8 (K) µg/g, respectively.

The influence of concomitants on the Ca, K, Mg, and Se determination by ICP OES was investigated through an addition and recovery test. The recovery showed the absence of matrix influence on the total Ca, K, Mg, or Se determination with recoveries ranging from 95% (Ca) to 100% (Se). According to U.S. Food & Drug Administration (FDA) guidelines for elemental determination by spectrometric techniques, the recovery tolerance for food samples should range from 80% to 120%^21^.

The total content was obtained in previous work done by our research group^22^. The total content of Ca, K, Mg, and Se in the pink and white oyster mushrooms (control and enriched groups) is shown in Table 1. It was observed that the two mushroom species were able to absorb Se(IV) and to accumulate Se in the fruiting bodies, especially the pink oyster mushroom. For pink and white mushrooms, the K and Mg content did not suffer alteration with the enrichment procedure, but the Ca concentration decreased 35% (pink oyster mushroom) and 28% (white oyster mushroom) compared to the control group.

**Table 1:** Total concentration of Ca, K, Mg, and Se in control and Se-enriched groups of the pink oyster and white oyster mushrooms.

	Total concentration ± standard deviation (n = 3)			
	pink oyster mushroom		white oyster mushroom	
	Control	Se-enriched	Control	Se-enriched
Ca (µg/g)	31 ± 1^a^	20 ± 1^b^	34 ± 2^a^	25 ± 2^b^
K (mg/g)	19.5 ± 0.4^a^	20.4 ± 0.3^a^	14.8 ± 0.4^a^	15.8 ± 0.2^a^
Mg (mg/g)	2.7 ± 0.1^a^	2.5 ± 0.2^a^	1.6 ± 0.1^a^	1.7 ± 0.1^a^
Se (µg/g)	<LOQ^a^	76 ± 2^b^	<LOQ^a^	48 ± 2^b^

Different superscripts (a,b) between the control and Se-enriched groups indicate significant differences between the elemental concentrations (p < 0.05).

It is important to point out that knowing only the total content of the essential elements in the mushrooms cannot reveal biological and metabolic information about these fungi, which is an imperative achievement for elemental distribution studies. Therefore, the imaging tools are essential to explain analytical issues^11^, as described by the proverb, “One picture is worth ten thousand words”.

### Elemental mapping of mushrooms

In elemental distribution mapping by LIBS, laser pulses are focused on the sample surface and the ablation, atomization, and excitation steps occur simultaneously during a single laser pulse^11^. The intensity of the various emission lines corresponding to the specific elements are directly related to its quantity in the sample^12,14,23^. In order to obtain elemental distribution mapping images, sample surface scans are performed on the regions of interest, in which the laser-induced plasmas are continuously generated and the elemental mapping profiles can be obtained from the analytical signal intensities, spectra, or concentrations of the elements of interest^11^.

**Figure 1:**
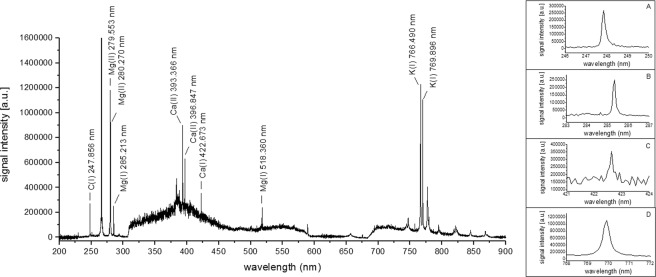
LIBS spectrum and individual signals for (**A**) carbon, (**B**) magnesium, (**C**) calcium, and (**D**) potassium of the pink oyster mushroom (accumulated signals from 10 laser shots).

**Figure 2:**
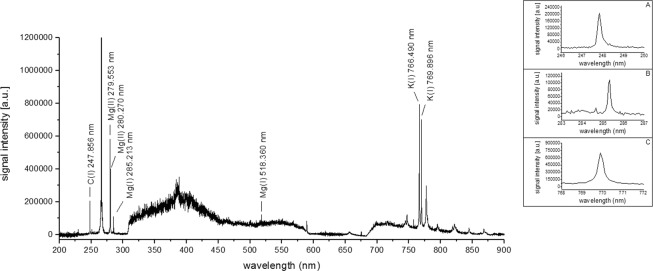
LIBS spectrum and individual signals for (**A**) carbon, (**B**) magnesium, and (**C**) potassium of the white oyster mushroom (accumulated signals from 10 laser shots).

During LIBS optimization, the highest analytical signal intensities and SBR of K (K(I)769.896 nm) were obtained using the instrument conditions shown in Table 2 for 36 mm^2^ of a representative area of the pink and white oyster mushroom samples (control group). The LIBS spectra of the pink and white oyster mushrooms are presented in Figs 1 and 2, respectively. For the pink oyster mushroom, C, Ca, Mg, and K were identified, while only C, K, and Mg were identified for the white oyster mushroom. Therefore, mapping of these elements and the evaluation of the Se enrichment effects on their distribution using LIBS imaging was shown to be feasible.

**Table 2:** Instrument parameters for elemental mapping of Ca, K, and Mg by LIBS.

Air flow (L/min)	Absent
Energy per pulse (mJ)	20
Spot size (µm)	50
Repetition rate (Hz)	10
Accumulated laser pulses	10
Delay time (µs)	0.15
Gate width (ms)	1.05
Pattern analysis	Site-to-site
Distance between craters (mm)	1
Analytical wavelength – atomic line	Ca(I) 422.673 K(I) 769.896 Mg(I) 285.213 C(I) 247.856

Although there were no significant differences in the total Ca concentration between the control groups, the Ca analytical signals in the LIBS spectrum were observed only for the pink oyster mushroom. This can be related to differences in matrix compaction between the two mushrooms that may change the analytical signal^13^. The porosity and low compaction of the mushroom matrix can modify the laser-sample interaction and, consequently, the sensitivity, because the signals can be attenuated by the noise during its acquisition. Thus, despite the fact that the Ca concentrations obtained by ICP OES are similar between the control groups, the higher porosity and lower matrix compaction of the white oyster mushroom may have prevented the detection of the Ca signal by the LIBS system. Furthermore, despite the similar levels of K and Mg between the control groups, it can be seen in Figs 1 and 2 that both elements presented smaller signal intensities for the white oyster mushroom, providing evidence of the effect of porosity and compaction of the matrix. However, evaluation of the matrix compaction was not done because it was outside the scope of this study.

**Figure 3:**
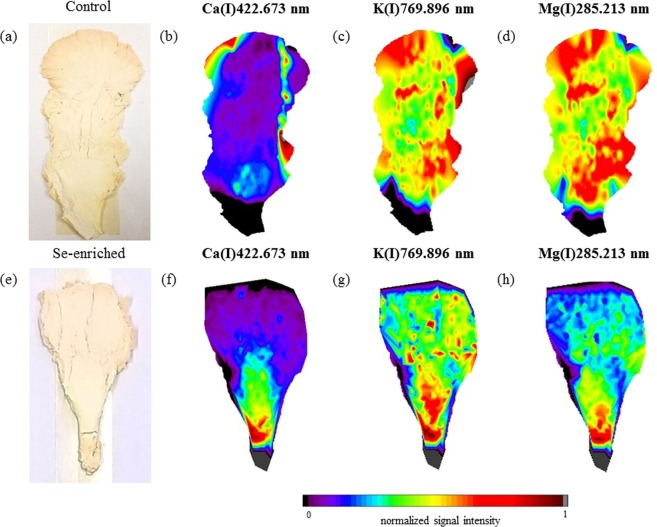
Fruiting body (**a**) and elemental distribution map of Ca (**b**), K (**c**), and Mg (**d**) in the control group, and fruiting body (**e**) and elemental distribution map of Ca (**f**), K (**g**), and Mg (**h**) in the Se-enriched pink oyster mushroom.

**Figure 4:**
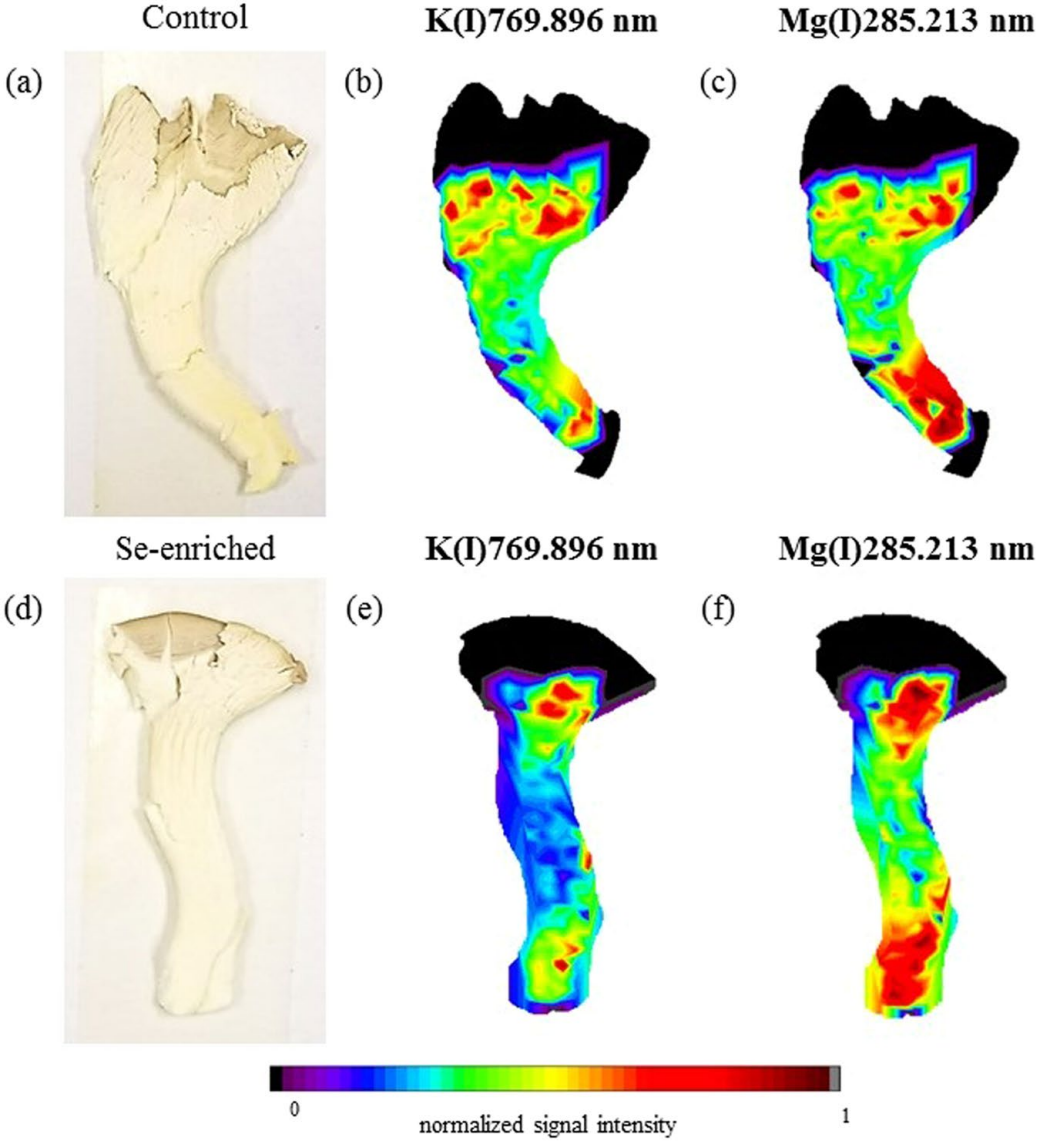
Fruiting body (**a**) and elemental distribution map of K (**b**) and Mg (**c**) in the control group, and fruiting body (**d**) and elemental distribution map of K (**e**) and Mg (**f**) in the Se-enriched white oyster mushroom.

**Figure 5:**
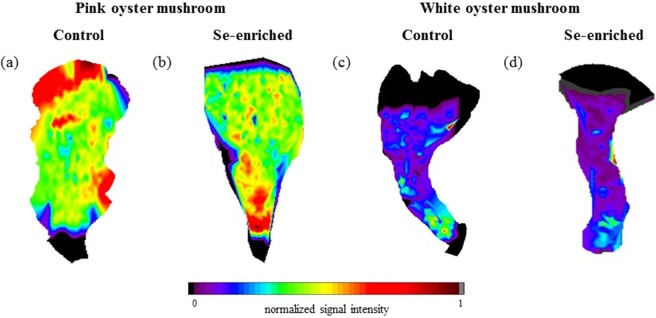
Elemental distribution map of C in the control (**a**) and Se-enriched (**b**) pink oyster mushroom, and control (**c**) and Se-enriched (**d**) white oyster mushroom.

Pictures of the mushroom fruiting bodies and distribution maps of Ca, K, and Mg (pink oyster mushrooms) and K and Mg (white oyster mushrooms) are shown in Figs 3 and 4, respectively. In the pictures of both species of mushrooms it is evident that the Se enrichment altered the morphological characteristics and elemental distribution of the fruiting bodies. The C distribution maps for the two mushroom species and culture conditions are shown in Fig. 5. The colour scale represents the normalized signal intensity of C, Ca, K, or Mg at sites of the mushroom fruiting bodies analysed by LIBS. The normalization of the signals for each element was determined from the ratio of the intensity of the individual signal and the highest intensity signal on the map as a whole. It was possible to verify that although C is homogeneously distributed in the mushrooms, some areas showed higher signal intensities, mainly for the pink oyster mushroom, indicating probably higher or lower tissue density in the respective regions and possible variations in the mass ablated by the laser pulse. To overcome this difficult, an useful approach is the use of a carbon emission line as internal standard to correct the variations in the spectra caused by sample surface uniformity, physical interference (porosity and matrix compaction), and variations in the mass ablated by laser pulses along the fruiting bodies^13^. Thus, the elemental distribution mapping using the emission signal of C(I)247.856 nm as internal standard was also evaluated.

**Figure 6:**
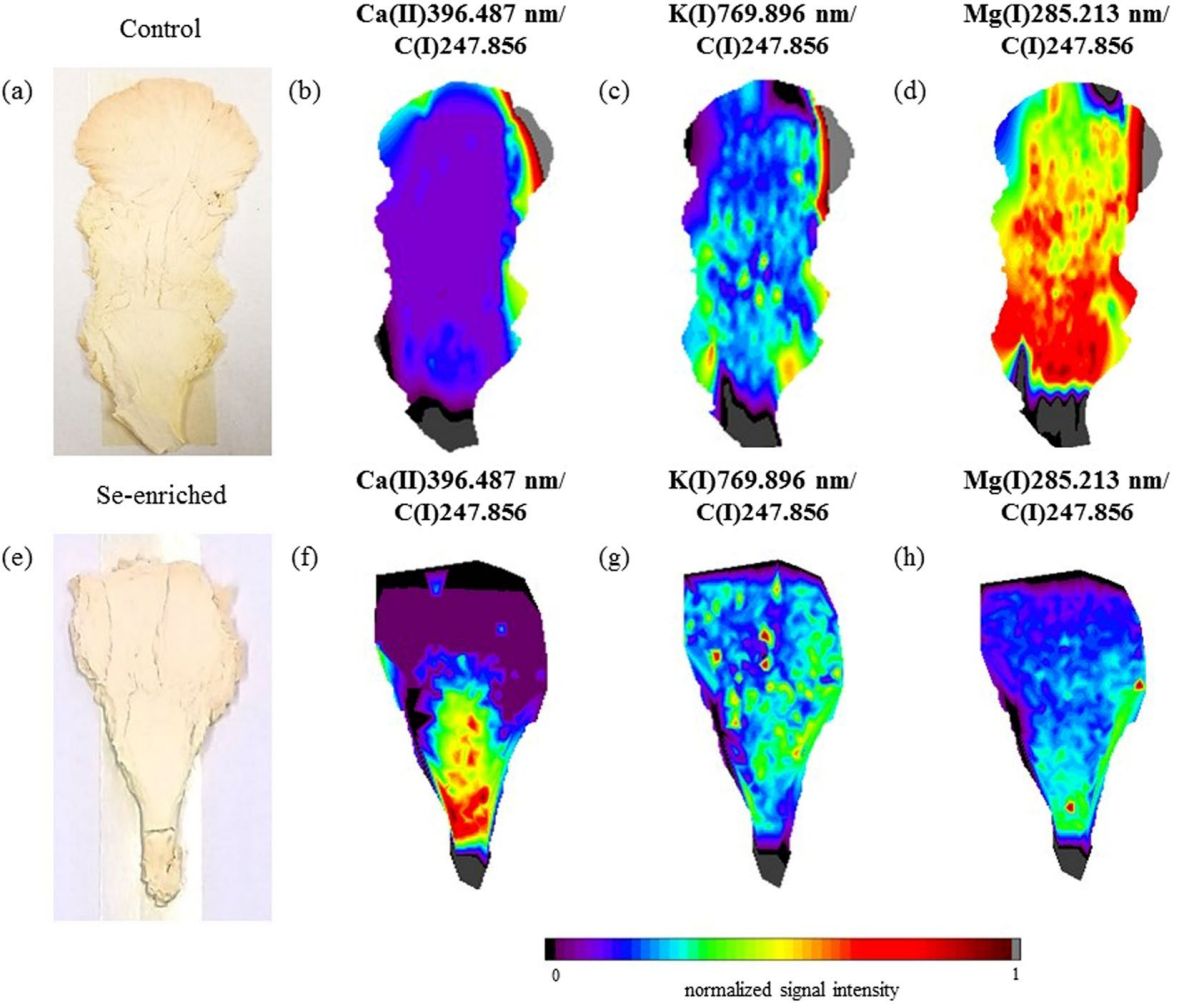
Fruiting body (**a**) and elemental distribution map of Ca (**b**), K (**c**), and Mg (**d**) in the control group, and fruiting body (**e**) and elemental distribution map of Ca (**f**), K (**g**), and Mg (**h**) in the Se-enriched pink oyster mushroom using C(I)247.856 nm as an internal standard.

**Figure 7:**
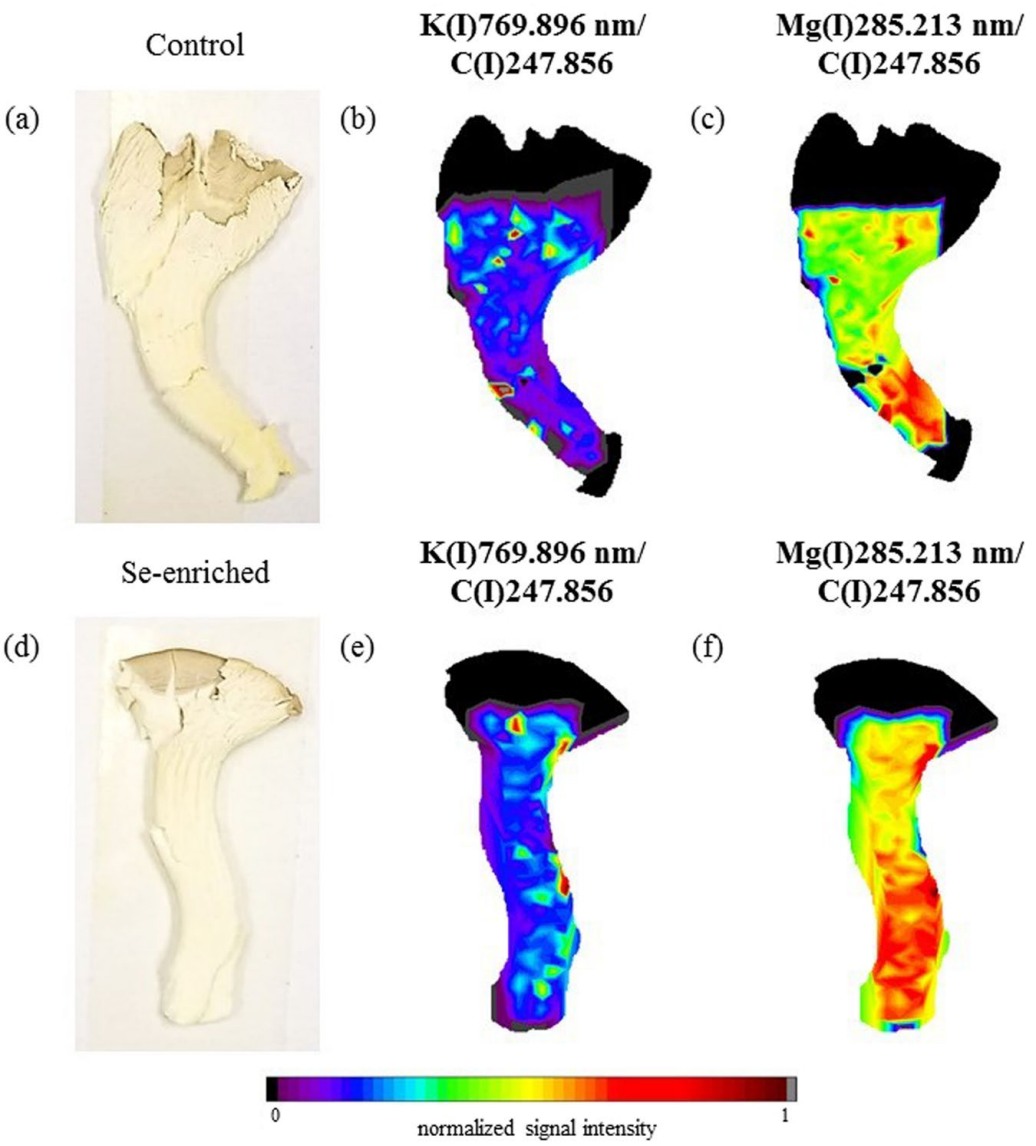
Fruiting body (**a**) and elemental distribution map of K (**b**) and Mg (**c**) in the control group, and fruiting body (**d**) and elemental distribution map of K (**e**) and Mg (**f**) in the Se-enriched white oyster mushroom using C(I)247.856 nm as an internal standard.

The distribution maps of Ca, K, and Mg (pink oyster mushrooms) and K and Mg (white oyster mushrooms) using C as an internal standard are shown in Figs 6 and 7, respectively. In general, although elemental maps with more homogeneous distributions were obtained with this method, the use of C as an internal standard did not change the elemental distribution and accumulation trends of most elements in specific areas of the fruiting bodies for different species of mushrooms under the determined cultivation conditions (control and enriched groups). That is, it was verified that in specific sites of the fruiting bodies, the high signal intensities altered the colour of the areas, but the elements’ accumulation trend was maintained, except for Mg and K in the Se-enriched pink oyster mushroom.

The black areas at the lower and upper edges of the elemental mapping images (Figs 3–7) correspond to the unmapped areas. Due to the high porosity and low tissue density of the unmapped areas in the fruiting bodies, fragmentation of the material occurs with the incidence of laser pulses. Since LIBS analytical outcomes can be influenced by sample fragmentation and re-deposition of particles^24^, the application of the instrumental parameters presented in Table 2 precluded the mapping of elemental distribution in these edge regions.

The Ca distribution mapping of the control group of the pink oyster mushroom (Figs 3b or 6b) indicates that this element is homogeneously distributed along the fruiting body at the lower levels of the intensity colour scale, except in the upper and lower right edges. However, in the Se-enriched pink oyster mushroom (Figs 3f or 6f), a higher intensity of Ca is evident in the lower part of the fruiting body and decreases in the upper part, indicating that in addition to Se enrichment promote a decrease in Ca concentration (Table 1), it led to an accumulation of Ca in the lower part of the mushroom, preventing Ca transport to the edges and upper part of the mushroom.

In the pink oyster mushroom (control group) the distribution maps of K (Fig. 3c) and Mg (Fig. 3d) showed that these elements exhibit similar distribution with good numerical correlation (R² = 0.7148). From a biological point of view, these two elements have similar functions in the fungal organism, both of which play a key role in the enzyme system. While K is required for protein synthesis and as a cofactor in many enzymatic reactions in cells, Mg is required by several enzymatic systems, including the activation of those involved in the metabolism of adenosine triphosphate (ATP)^25^.

Although the Se enrichment did not change the total mass fraction of Mg in the pink oyster mushroom (Table 1), the distribution of Mg (Fig. 6h) was altered. After the enrichment there was a more homogeneous distribution of Mg compared with the control group (Figs 3d or 6d) and a decrease in the numerical correlation between the K (Fig. 6g) and Mg (Fig. 6h) distribution (R² = −0.0129). The maps shown in Figs 3h and 6h indicate that Mg accumulated in the lower part of the fruiting body and the elemental transport was inhibited. For the distribution of K in the Se-enriched pink oyster mushroom (Fig. 6g) it was verified that there was an accumulation of K in central sites, indicating that the transport was less inhibited compared with the Mg map (Fig. 6h). The decrease in the numerical correlation between Mg and K in the Se-enriched pink oyster mushroom compared with the control group indicates that Se enrichment changed the biochemical processes of these elements in fungi metabolism.

The elemental imaging in the white oyster mushrooms indicated that the Se enrichment did not significantly alter the K (Fig. 4b,e) and Mg (Fig. 4c,f) distributions compared to the control group. Both elements accumulated in the upper and lower areas of the fruiting bodies under both cultivation conditions. The Mg and K distributions in the white oyster mushroom control group show a good numerical correlation (R² = 0.5851), and after Se enrichment, the numerical correlation between Mg and K (R² = 0.7828) increased, indicating that the Se enrichment may have favoured possible synergistic reactions between Mg and K in the metabolism of this species of mushroom. Note that the total K and Mg concentrations increased with the enrichment procedure (Table 1).

Finally, it is important to point out that Se determination and/or distribution by LIBS could also add information about Se enrichment effects. However, the Se detection and/or determination is not a trivial task, because the intensities of Se emission lines are weak and close to deep ultraviolet (UV) region (close to 200 nm). In addition, the Se emission lines are resonance lines, which can be affected by self-absorption in cases of samples with high Se concentration^26^.

## Conclusions

This study demonstrated the feasibility and capability of using LIBS imaging, a fast-analytical method (without sample preparation step), to evaluate the uptake and transport of Ca, Mg, and K in edible mushrooms. It also demonstrated the effects of Se enrichment on the distribution of these macroelements, as well as possible elemental competitions, through elemental distribution mapping. The elemental distribution mapping made it possible to visualize the accumulation trends of different elements in specific areas of the mushroom fruiting bodies, which may be of interest in understanding interactions between the fungi and their environment.

It was verified that although enrichment does not modify the total concentration of some elements, it can alter the elemental distribution in the mushroom fruiting bodies. The procedure of Se(IV) enrichment changed the distribution of Ca, Mg, and K primarily in the pink oyster mushroom, indicating that the strategy of enrichment with Se commonly applied to edible mushrooms alters the transport and compartmentalization of these elements, which may be biologically critical with respect to the fungal metabolism.

## Methods

### Instrumentation

The elemental distribution mapping was carried out with a J200 Tandem LIBS system (Applied Spectra, CA, USA) with a Nd:YAG laser operating at 266 nm (laser beam diameter of 4 mm, 20 mJ per pulse, and pulse duration of 6 ns). The LIBS system contained a 6 channel-CCD spectrometer (Applied Spectra, USA) with a spectral range from 186 to 1044 nm. Analyses were performed using the instrument parameters shown in Table 2.

The quantification of total Ca, K, Mg, and Se was done by inductively coupled plasma optical emission spectrometry (ICP OES) using an iCAP 6300 Duo spectrometer (Thermo Fisher Scientific, England) equipped with axial and radial plasma view configurations. A charge-injection device detector was used, which allows measurements from 166 to 847 nm^27^. An echelle polychromator was purged with argon, and a radiofrequency source of 27.12 MHz was used. The instrument parameters for the ICP OES analysis are given in Table 3.

**Table 3:** Instrument parameters and heating program for Se determination by ICP OES.

Power	1250 W
Nebulizer	Meinhard
Spray chamber	Cyclonic
Plasma gas flow	15 L min^−1^
Intermediate gas flow	1.0 L min^−1^
Nebulizer gas flow	0.45 L min^−1^
Sample flow rate	1.5 mL min^−1^
Analytical wavelength (axial view) – atomic line (I) or ionic line (II)	Ca(II)^b^ = 318.1 K(I)^a^ = 769.8 Mg(II)^b^ = 279.0 Se(I)^a^ = 196.0

The mushrooms species were dried in a freeze dryer (Thermo Fisher Scientific, England). Before Ca, K, Mg, and Se determination by ICP OES, the samples were submitted to digestion in a thermostatic water bath (model Q226M2, Quimis, Brazil).

### Reagents and samples

The substrates, composed of Brazilian sugarcane bagasse, rice bran, wheat bran, calcium oxide, and inoculated with pink or white oyster mushroom seeds, were obtained from a mushroom producer in Mairipora (Sao Paulo, Brazil).

All solutions were prepared using analytical reagent-grade chemicals, with high-purity deionized water obtained from a Milli-Q water purification system (Millipore, USA) as the solvent. Analytical grade 65% (w/v) HNO_3_, distilled in a quartz sub-boiling still (Marconi, Brazil), and 30% (w/v) H_2_O_2_ (Merck, Germany) were used for sample digestion in a thermostatic bath.

Titrisol standard solutions at concentrations of 1000 mg/L (Merck, Germany) for Ca (CaCl_2_), K (KCl), Mg (MgCl_2_), and Se (SeO_2_) were diluted in HNO_3_ to 0.1% (v/v) and used to prepare the reference analytical solutions for elemental determination by ICP OES.

Selenium solutions were prepared for the enrichment procedure using Na_2_SeO_3_ (Merck, Germany) at a concentration of 1121 mg/L.

### Cultivation of Se-enriched mushrooms

Mushroom cultivation was carried out in glass containers containing an organic substrate (20 g) based on Brazilian sugarcane bagasse composed of rice bran (5% w/w), wheat bran (5% w/w), and CaO (4% w/w) inoculated with the fungi seeds of *Pleurotus ostreatus* (white) or *Pleurotus djamor* (pink).

The mushrooms were cultivated with deionized water added to the inoculated substrate (control group) or the Se-enriched inoculated substrate. The production of Se-enriched mushrooms was done by adding 5 mL of Na_2_SeO_3_ solution at a concentration of 1121 mg/L to the inoculated substrates to obtain 25.6 µg/g of Se(IV) on the inoculated substrate of pink and white oyster mushrooms.

The inoculated substrates were incubated in a portable greenhouse under dark conditions and temperatures ranging from 23 to 25 °C for a total incubation period of 7 days. The relative humidity of the air was then raised to 80% until the formation of the mushroom fruiting body shortly afterwards. During the incubation and fruiting period, deionized water was sprayed 3 times daily on the different culture groups. The total culture period was 13 days for all cultivation conditions of both mushroom species.

The mushrooms were collected, washed with deionized water, and freeze-dried for 48 h before performing the LIBS elemental mapping analysis. For total elemental quantification by ICP OES, the dried samples were frozen in liquid nitrogen and then ground using a mortar and pestle. The different groups and species of mushrooms were stored in polypropylene tubes and kept frozen at −4 °C.

### Sample digestion and total Ca, K, Mg, and Se determination by ICP OES

For total Ca, K, Mg, and Se determination by ICP OES, the samples were first submitted to an acid digestion procedure in a thermostatic bath, by adding 2 mL of HNO_3_ to 30 mg of samples (control group and Se-enriched mushrooms) and keeping the mixture at 80 °C for 1 h. Thereafter, 1 mL of H_2_O_2_ was added and the mixture was maintained at 80 °C for 30 min. After digestion, the volume of the solutions was increased to 10 mL with deionized water.

The digested samples were analysed by ICP OES after method calibration using analytical solutions with concentrations ranging from 10 to 100 mg/L (Ca, K, and Mg) or 0.5 to 5.0 mg/L (Se) in 20% (v/v) HNO_3_. The interest elements determination was done in robustness conditions of the spectrometer and in the absence of internal standard. The limit of detection (LOD) was calculated in accordance with the International Union of Pure and Applied Chemistry (IUPAC) recommendations, using the background equivalent concentration (BEC) and signal-to-background ratio (SBR)^27^. Thus, BEC = C_rs_/SBR; SBR = (I_rs_ − I_blank_)/I_blank_; LOD = 3 × BEC × RSD/100; where C_rs_ is the concentration of the multi-elemental reference solution, and I_rs_ and I_blank_ are the emission intensities for the multi-elemental reference and blank solutions, respectively. The limit of quantification (LOQ) was calculated as 10 times the LOD^28,29^.

The chemical interferences in the elemental determination were evaluated using the addition (20.0 mg/L (Ca, Mg, and K) and 1.0 mg/L (Se)) and recovery test. The analytical solutions of the interest analytes were added before the acid digestion procedure.

### Elemental imaging by LIBS

The analyses were conducted at room temperature and pressure. The experimental conditions were adapted from studies published by our research group using the same LIBS system^30^. Optimizations of the argon flow, delay time (0, 0.10, 0.15, 0.20, and 0.30 µs), spot size (35, 50, 65, 85, 100, 120, and 140 µm), and number of pulses (10, 20, and 50) were performed, monitoring the analytical signal intensities and the signal-to-background ratio (SBR) of K, using the wavelength K(I)769.896 nm, for both mushroom species of the control group.

The craters number was selected to ensure complete coverage of the fruiting bodies of the mushrooms. The sample surfaces were scanned by the laser beam using a site-to-site pattern. During the sample scan, before the laser pulses were started at each position of the sample from the site-to-site pattern, the auto-focus system was automatically adjusted according to the material surface inclination, allowing it to compensate for any flatness anomalies and to accurately control the focalization distance (the objective-to-sample anomalies).

The emission lines of the spectrum ranged from 190 to 1044 nm and were identified using the NIST (2013) spectroscopy database^31^ and Aurora software (Applied Spectra, USA). For background (BG) correction by the equipment software, the BG average of the regions surrounding each selected emission line was calculated and subtracted from the maximum intensity of each emission line.

The colour scale of the pictures of the mushroom fruiting bodies and the distribution maps of C, Ca, K, and Mg in the pink and white oyster mushrooms were associated with the normalized analytical signal intensities of C, Ca, K, and Mg, in which the normalization of the signals for each element was determined from the ratio between the intensity of the individual signal of each element and highest C(I) 247.856 intensity signal obtained on the map as a whole. Additionally, the C(I) 247.856 emission line of the sample itself had the function of an internal standard.

The elemental images were constructed by considering each point on the sample surface represented by the analytical signal intensity and using a false-colour scale to present a visual result. All images were initially processed using ImageJ software (NIH, Bethesda, USA); the images were constructed with the find edges resource to facilitate highlighting the crater positions in the samples. Thereafter, the different points on the sample surface were selected to obtain the x-y coordinates. Finally, the multi-colour maps were created using Origin 8 software (OriginLab Corp., Northampton, USA), assigning an arbitrary colour scale on the sample scanning surface over the region of interest where the x-axis and y-axis represented the coordinates (location) of the laser shots and the z-axis represented the signal intensity for each element.

### Statistical analysis

Acid digestion of pink and white oyster mushrooms (control and Se-enriched groups) was carried out in triplicate and all measurements were conducted on the three replicate samples. The mean concentrations of Ca, K, Mg, and Se with significant statistical differences (p < 0.05) between the control group and the enriched mushrooms of the two species were evaluated by using Student’s *t*-test.
